# Synchronous Penile Metastasis from a High-Grade Adenocarcinoma of the Prostate

**DOI:** 10.1155/2012/193787

**Published:** 2012-10-16

**Authors:** S. Dijkstra, A. G. van der Heijden, H. E. Schaafsma, P. F. A. Mulders

**Affiliations:** ^1^Department of Urology, Radboud University Nijmegen Medical Centre, Geert Grooteplein Zuid 10, P.O. Box 9101, 6500 HB Nijmegen, The Netherlands; ^2^Department of Pathology, Radboud University Nijmegen Medical Centre, Geert Grooteplein Zuid 10, P.O. Box 9101, 6500 HB Nijmegen, The Netherlands

## Abstract

Metastasis to the glans penis is a rare phenomenon and usually occurs in a late stage of disease. A 68-year-old man was referred to our clinic because of two indurated lesions of the glans penis and minor lower urinary tract symptoms. Digital rectal examination revealed a hard nodular prostate, and serum prostate-specific antigen (sPSA) level was 13.3 ng/mL. Biopsies of the penile lesions and transrectal ultrasound-guided prostate biopsies were taken. Immunohistochemical staining of formalin-fixed paraffin-embedded tissue exposed a synchronous penile metastasis from a high-grade adenocarcinoma of the prostate. Except a pathologically enlarged lymph node detected with MRI there was no suspicion on other metastases. Currently this patient is being treated with a Gonadoreline (GnRH) antagonist. Nevertheless, the prognosis will be poor.

## 1. Introduction

Prostate cancer is the most frequent solid malignancy diagnosed in the Western male population, and the incidence is still rising [1]. An estimated 8% of all prostate cancers is diagnosed in an advanced stage of disease [2]. Metastases to the penis are a rare phenomenon, and the prognosis is usually very poor [3].

This paper deals with a 68-year old patient diagnosed with synchronous penile metastases from a high-grade prostate carcinoma, without distant metastasis, a case never been described in literature before.

## 2. Case Presentation

**Figure 1:**
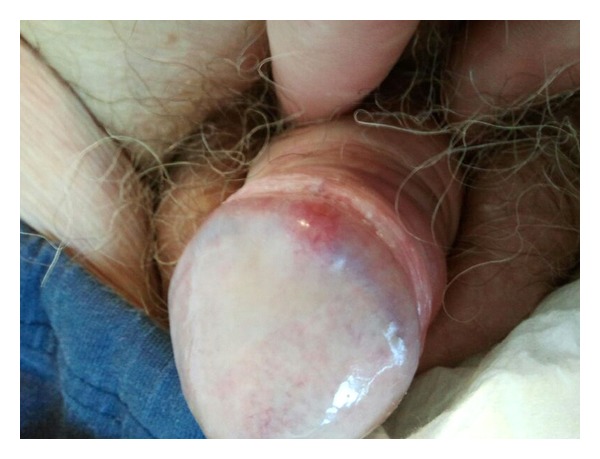
Focal indurated erythema on the dorsal side of the glans penis.

A 68-year-old man was referred to our clinic in December 2011, because of an abnormality of his glans penis. The patient noticed the abnormality two weeks before consulting the urology department of a community hospital as a red, painless, and elevated lesion on the dorsal side of the glans penis (Figure 1). Physical examination revealed an indurated lesion with a diameter of 14 millimeter. There were no signs of enlarged inguinal lymph nodes. This has been confirmed by ultrasound of the inguinal region. A biopsy of the penile lesion was taken, which showed suspicion for malignancy. Since further specification was not possible, a second biopsy was taken. No abnormalities were found in the pathological specimen. Besides the above mentioned, the patient complained of lower urinary tract symptoms and therefore the serum prostate specific antigen (sPSA) level was determined. The sPSA level was elevated to 13.3 ng/mL. Transrectal ultrasound-guided prostate biopsies were taken, and the patient was referred to our clinic.

At physical examination also a second abnormality in the glans penis was observed. Digital rectal examination revealed an enlarged multinodular prostate.

**Figure 2:**
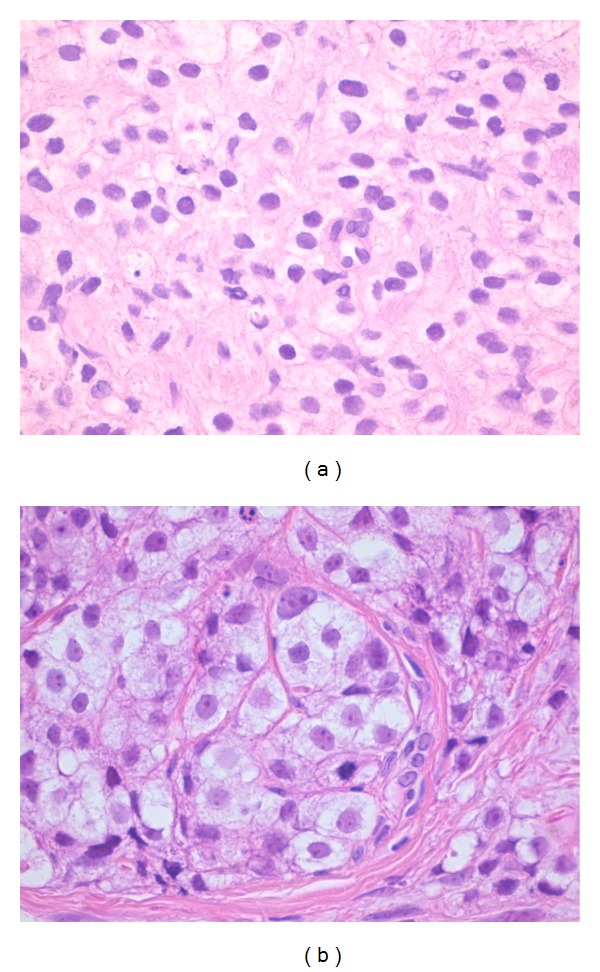
Haematoxylin-eosin stain of a poorly differentiated tumour from the prostate (a) and the pathological penile specimen (b).

**Figure 3:**
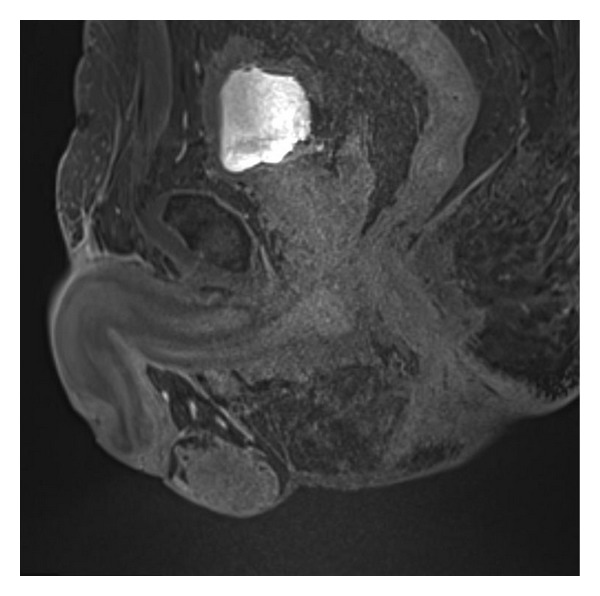
MRI scan which shows a focal lesion on the dorsal side of the glans penis.

Prostate biopsies showed a poorly differentiated adenocarcinoma of the prostate with Gleason score 10 (5 + 5). New biopsies from the penile lesion were taken and revealed a malignant cell type of unknown origin. Although immunohistochemical staining for PSA was negative, findings most likely matched the poorly differentiated prostate carcinoma (Figure 2). Subsequently, a computed tomography (CT) scan and bone scintigraphy were performed. The latter showed no suspicion of bone metastases. The CT scan revealed no abnormalities besides the earlier known prostate cancer. Also a magnetic resonance imaging (MRI) scan was carried out which presented a stage 4 prostate cancer, one pathologically enlarged lymph node and a focal lesion in the penis, suspect for a metastasis of prostate carcinoma (Figure 3).

Having been diagnosed as metastatic prostatic cancer, the patient is treated with a Gonadoreline (GnRH) antagonist.

## 3. Discussion

Prostate cancer is the most common malignancy in the male population, and despite various treatment options it progresses to a metastatic stage disease in many patients. Generally, it is known to metastasize to pelvic lymph nodes and bone. Less frequent also lung and liver can be affected. In a review Mueller et al. reported a total of 456 cases of cutaneous metastases from urologic malignancies, for which over 85% originated from kidney and bladder carcinoma as primary malignancy. Only 12% of the cutaneous metastases originated from the prostate. Overall, the incidence of cutaneous metastasis with the prostate as primary tumour site is less than 0.5% [4]. A metastasis in the penis is even less common. Chaux et al. described a total number of 437 cases with secondary penile tumours. Of these cases 133 (30.4%) originated from the prostate [5]. All cases of metastases from prostate cancer to the penis, described in this review, are metachronous. Although other primary tumour sites have been described as synchronous (lung, tongue, and sigmoid), to our knowledge, a synchronous clinical presentation of a penile metastasis from prostate cancer has never been described previously.

The penile shaft is the most common anatomical site to be affected in case of a penile metastasis. This is in contrast to the patient presented in our case, where only the glans penis was affected. Tumours limited to the glans penis occur less frequent and account for 24% of all penile metastases [5].

Although penile metastases are a very rare phenomenon, clinical presentation of a penile lesion should always be considered to be an expression of a disseminated disease with an extremely poor prognosis. In case of prostate cancer as primary malignancy, a cancer-related death varying from 4 to 18 months from the time of penile metastasis presentation must be taken into account [5].

Several metastatic mechanisms have been described in literature. Taking into consideration that the penis is a highly vascularized organ, hematogenous spread will be the mechanism of spread in this case. Therefore it is expected that penile metastasis of high-grade malignancy of the prostate is accompanied by other distant metastases. As described previously in our case, no other distant metastases were found.

Metachronous penile metastases from the prostate are mostly known to originate from a high-grade malignancy and have a very poor prognosis. Synchronous penile metastases from a prostate carcinoma, without any signs of distant metastases, have never been described before, but we presume that the prognosis of this patient will also be very poor.
